# An enigmatic tale of macrophages in bone marrow causing inflammation of the brain: A case report on CNS HLH

**DOI:** 10.1016/j.htct.2025.103981

**Published:** 2025-09-08

**Authors:** Ivy E. Verriet, Jessica Liu, Adrienne Fulford, Uday Deotare

**Affiliations:** a Department of Medicine, Schulich School of Medicine and Dentistry, Western University, London, ON, Canada; b Verspeeten Family Cancer Centre, London Health Sciences Centre, London, ON, Canada; c Department of Oncology, Schulich School of Medicine and Dentistry, Western University, London, ON, Canada; d The Centre for Quality, Innovation and Safety, Department of Medicine, Schulich School of Medicine and Dentistry, Western University, London, ON, Canada

**Keywords:** Allogeneic stem cell transplant, Central nervous system, Hemophagocytic lymphohistocytosis, Case report

## Abstract

**Background:**

Hemophagocytic lymphohistiocytosis (HLH) is a life-threatening immune disorder characterized by excessive inflammation and multiorgan involvement. Rarely, HLH can manifest with signs and symptoms isolated to the central nervous system (CNS). This case report highlights the unique clinical course of CNS-isolated HLH in a 19-year-old female who, despite a nine-year delay in diagnosis, achieved disease remission following a hematopoietic stem cell transplant (HSCT).

**Case:**

The patient initially presented at 9 years old with seizures, ataxia, and progressive cognitive decline. Over the next nine years, extensive diagnostic evaluations were performed, including neuroimaging, cerebrospinal fluid analysis, and genetic testing. Genetic testing identified a compound heterozygous mutation in the PRF1 gene, confirming a diagnosis of familial HLH (FHL). The patient underwent hematopoietic stem cell transplant (HSCT) from an HLA-matched unrelated donor. Despite significant complications, including multiple infections and renal failure, she achieved remission. Six years post-transplant, the patient exhibited stabilization of neurological function, cessation of seizures, and absence of active HLH.

**Conclusion:**

This case underscores the importance of considering genetic testing in patients with unexplained CNS symptoms and atypical radiological findings. Timely HSCT, even in cases with delayed diagnosis, can lead to remission and improved quality of life.

## Introduction

Hemophagocytic lymphohistiocytosis (HLH) is a life-threatening disorder of immune dysregulation, manifesting as either familial or secondary to infection, malignancy, or autoimmune disease. Familial HLH has been associated with mutations in four genes (PRF1, STX11, STXBP2, and UNC13D), which account for over 90 % of familial cases [1].

Diagnosis of HLH is currently based on the HLH-2004 criteria, which emphasizes systemic disease markers such as fever, splenomegaly and cytopenias [2,3]. Another frequent finding is central nervous system (CNS) involvement due to infiltration of activated lymphocytes and macrophages into the meninges and brain, which can present as seizures, encephalopathy, and cerebellar involvement on MRI [4]. From 30–73 % of patients with systemic HLH have CNS-HLH, but rarely patients will present with isolated CNS involvement [2,4].

Hematopoietic stem cell transplant (HSCT) is now recognized as the only curative option for HLH [3,5]. Numerous studies have demonstrated the effectiveness of HSCT in achieving long-term remission, however, delays in diagnosis, especially with CNS involvement, are associated with increased risk of relapse and poorer outcomes due to the cumulative irreversible CNS damage [3,4,6]. Few cases exist where HSCT has successfully cured CNS-isolated HLH years after initial presentation, with the longest documented period being seven years [7]. Here, we report a unique case of a 19-year-old girl with CNS-isolated HLH, successfully cured with HSCT nine years after symptom onset.

## Case presentation

In March 2009, a previously healthy nine-year-old girl presented with progressive neurological symptoms, including headaches, vomiting, and dizziness, and was subsequently admitted to the hospital with sudden-onset confusion, blindness, and incoherent speech. She had an unremarkable perinatal history and no significant personal or familial medical history.

Initial investigations included serological and cerebral spinal fluid (CSF) testing for opportunistic and routine viral infections, all of which were negative. Magnetic resonance imaging (MRI) showed diffuse white matter abnormalities and T2 hyperintensity around the optic nerves. Based on the clinical and radiological findings, a diagnosis of optic neuritis secondary to acute demyelinating encephalomyelitis (ADEM) was made, and the patient was treated with intravenous corticosteroids. A lumbar puncture revealed an opening pressure of 28 mmHg, necessitating the placement of an external ventricular drain, which was successfully removed after one week. The patient regained full vision and was clinically stable until May 2009, when she began to exhibit focal seizures and was started on clobazam. Over the next three months, her condition worsened, with gait disturbances, memory deterioration, and persistent focal seizures despite increasing doses of clobazam.

In early July, she was readmitted with encephalopathy, including a three-week history of increased lethargy, worsening memory, confusion, ataxia, and vomiting. MRI revealed new leptomeningeal enhancement with increased nodularity in the brain parenchyma. Two lumbar punctures performed during this hospitalization revealed only elevated protein (predominantly albumin) with a mild pleocytosis. All other investigations, including antinuclear antibody, oligoclonal banding, cryptococcal antigen testing, culture and cytology, testing were unremarkable. A repeat brain MRI just before discharge demonstrated significant improvement with the resolution of cortical lesions, although the patient continued to experience cognitive dysfunction and a wide-based gait.

**Figure 1:**
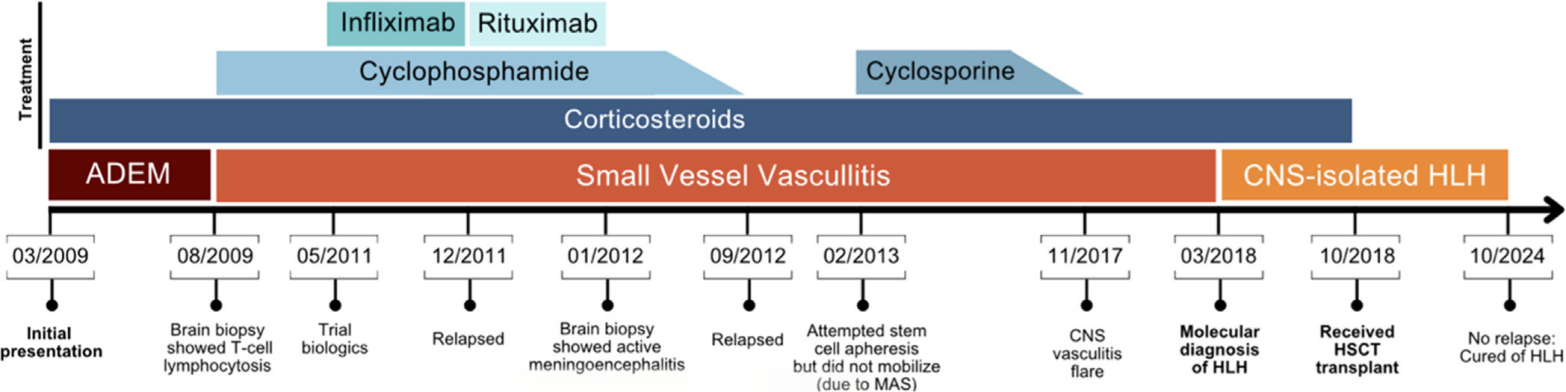
Timeline of patient course (not to scale). Running diagnosis indicated in shades of orange, medications administered to patient indicated in shades of blue. Corticosteroids were administered PO or IV sporadically throughout the disease course. Notable events during disease progression are dated and detailed below the arrow.

In August 2009, a brain biopsy revealed T-cell lymphocytosis surrounding small vessels, leading to a diagnosis of small vessel vasculitis. The patient was treated with prednisone, mycophenolate mofetil (CellCept), cyclophosphamide, and acyclovir. Figure 1 shows the timeline of the patient’s course over the next nine years, including multiple treatment modalities and relapses.

In March 2018, the patient underwent genetic testing which revealed a compound heterozygous mutation in the PRF1 gene, consistent with familial HLH. Further testing of her parents revealed the PRF1.886T>*C* (p.Tyr296His) mutation in her father, and the PRF1.481A>*G* (p.Lys161Glu) mutation in her mother. Given that the PRF.886T>*C* (p.Tyr296His) mutation was known to be pathogenic for HLH, she was referred for a potential curative HSCT.

**Figure 2:**
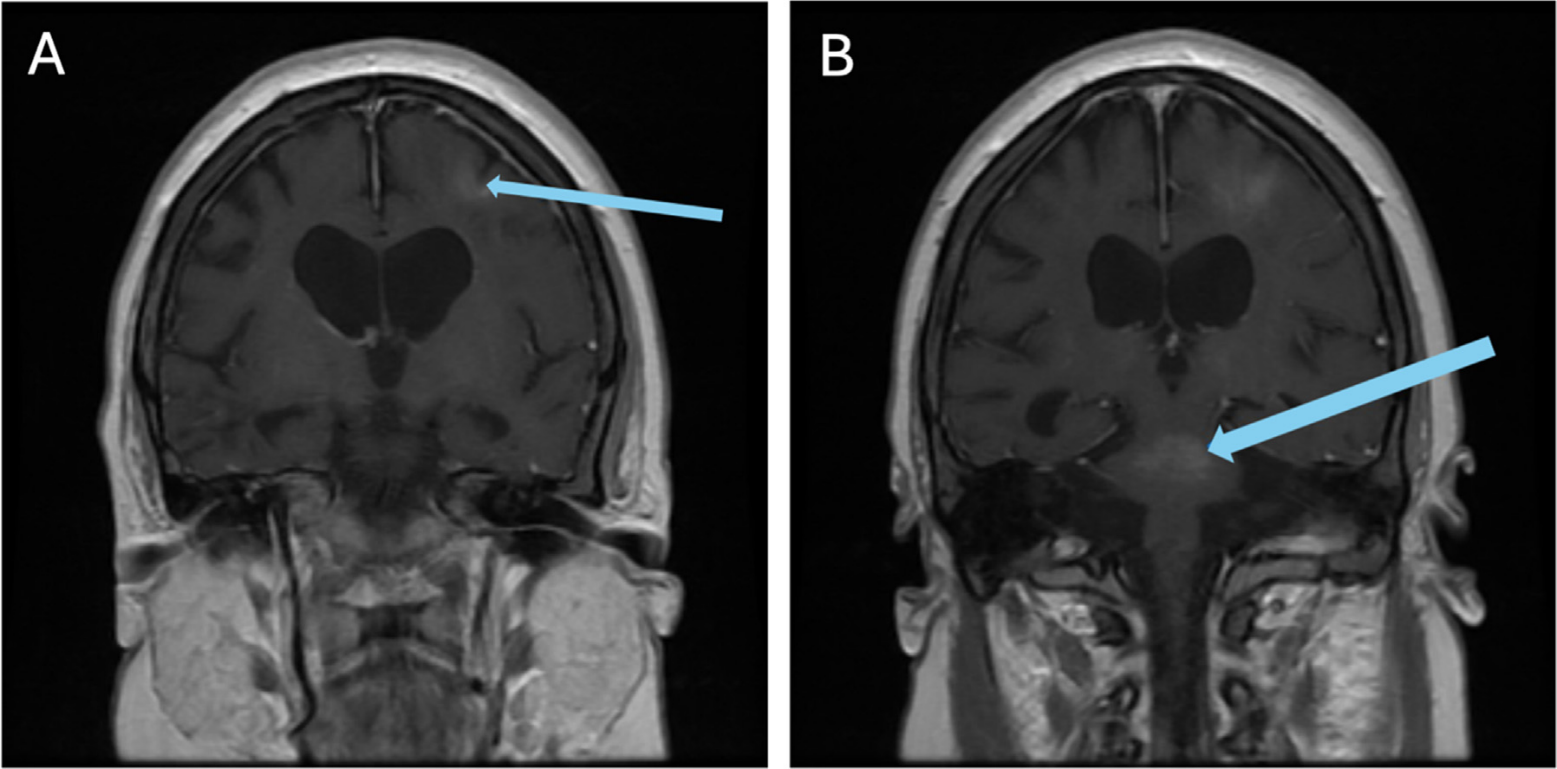
Pre-transplant magnetic resonance images demonstrating low grade enhancement in the precentral gyrus of the left frontal lobe extending in deep aspects of cortex asymmetrically (A) and stippled enhancement in dorsal aspect of mid pons (B) on post-gadolinium images.

In October 2018, the patient underwent an allogeneic HSCT from a 10/10 HLA-matched unrelated donor. A pre-transplant MRI was performed one month before transplant that found no enhancements to suggest any active disease (Figure 2). She underwent pre-transplant conditioning with fludarabine, treosulfan, and cytarabine (Cytarabine). Neutrophil and platelet engraftment took place 15 days post-transplant. Tacrolimus, rabbit anti-thymocyte globulin, methylprednisolone, and methotrexate (Methotrexate) were given for graft-versus-host disease (GvHD) prophylaxis. She developed acute GvHD (overall Grade I) of the skin and gut, which resolved with topical and systemic steroids. Chimerism 60 days post-transplant showed >95 % donor cells, and at 280 days post-transplant the patient demonstrated stable chimerism with CD3 donor cells at 97.4 %, CD19 at 98.9 %, and myeloid cells at 98 %, and remained at these levels thereafter.

Unfortunately, her post-transplant course was complicated by multiple infections including Epstein–Barr virus (EBV) viremia, varicella infection, and adenoviremia one-month post-transplant. She also experienced acute kidney injury secondary to tacrolimus and cidofovir toxicity that progressed to acute-on-chronic renal failure due to sepsis requiring hemodialysis in March 2019, and eventually a related living donor kidney transplant in January 2023.

Despite these complications, her neurological symptoms significantly improved. She experienced two seizures within the first-year post-transplant but has since been seizure free. Despite ongoing dystonic movements (which predated her transplant) for which she is on trihexyphenidyl, her cognitive function has returned to baseline. MRI findings one-year post-transplant showed no active disease, and she has been deemed cured of HLH. She is now six years post-transplant and is doing well, with no recurrence of disease or neurological symptoms.

## Discussion

Isolated CNS symptoms in the absence of systemic manifestations makes differentiating CNS-HLH from other CNS inflammatory diseases, such as ADEM, extremely challenging, as seen in this case. A study conducted by Deiva et al. compared radiological presentations of CNS-isolated HLH and ADEM and found that while both may present with white matter hyperintensity on MRI, CNS-HLH often features symmetrical, periventricular lesions, whereas ADEM typically involves the brainstem [8]. Our patient had asymmetrical white matter lesions and brainstem involvement, demonstrating that additional studies are necessary to fully differentiate these conditions (Figure 2).

The challenges of diagnosing CNS-isolated HLH are further highlighted by Benson et al., who describe three cases of CNS-isolated HLH initially misdiagnosed as ADEM or small vessel vasculitis [7]. Similar to our case, the diagnosis was ultimately confirmed through genetic testing. This case, in addition to those of Benson et al., underscores the importance of prompt genetic testing in patients with unexplained neurological symptoms and abnormal neuroimaging which would allow for earlier initiation of curative interventions like HSCT.

Although HSCT is widely known to be curative for systemic HLH, its role in CNS-isolated HLH is less established [3]. Previous cases have shown that early HSCT can halt disease progression and offer a cure when performed soon after symptom onset [7,9,10]. Our patient was unique in that she underwent HSCT nine years after her initial symptom onset. Despite the delay, her post-transplant MRI findings showed no further disease activity, although existing lesions and some neurological symptoms remain unchanged. This aligns with reports emphasizing the irreversible nature of CNS damage when treatment is delayed. Nevertheless, at six years post-transplant, our patient exhibits no further disease progression and is now considered cured of her CNS-HLH. This suggests that HSCT can still offer significant benefits, even after prolonged disease progression.

In conclusion, genetic testing for HLH-associated mutations should be pursued in patients with refractory inflammatory CNS disease and neurological impairment, as timely HSCT can halt disease progression, improve quality of life and provide curative outcomes, even after years of ongoing symptoms

## Ethics approval and consent to participate

Ethics approval is not required at our institution for individual cases or case series.

## Consent for publication

Verbal, informed and written consent was obtained from the patient through a legally authorized representative for anonymized patient information.

## Availability of data and materials

The authors confirm that the data generated and analyzed in this study are included in this published article.

## Clinical trial number

Not applicable

## Authors' contributions

IEV collected the raw patient data and wrote the manuscript. JL also collected patient data, designed the study and contributed to writing and editing the manuscript. AF provided guidance and obtained patient consent. UD designed the study and contributed to writing and editing of the manuscript with overall supervision. All authors read and approved the final manuscript.

MAS: macrophage activation syndrome; ADEM: acute demyelinating encephalomyelitis; HSCT: Hematopoietic stem cell transplant

## Funding

The authors did not receive any financial support for the purpose of this case report.

## Conflicts of interest

The authors do not have any competing interests to declare.
